# Resilient Cultural Practices in Mexican Immigrant Families: Children’s Helping and Parental Goals and Aspirations

**DOI:** 10.3390/bs15010050

**Published:** 2025-01-08

**Authors:** Angélica López-Fraire, Maricela Correa-Chávez

**Affiliations:** 1 Child Development Department, California State University, Dominguez Hills, Carson, CA 90747, USA; 2 Psychology Department, California State University, Long Beach, CA 90840, USA; maricela.correa@csulb.edu

**Keywords:** children’s helping, cultural practices, learning by observing and pitching in, parental goals

## Abstract

This study examined 20 parental interviews of third-grade children in U.S. Mexican-heritage families in California, focusing on their children’s helping at home, parents’ goals for their children, and the values they hoped to instill in their children. The families varied in their experience with Learning by Observing and Pitching In (LOPI), a way of organizing learning that is consistent with the traditions of Indigenous and Indigenous-heritage communities of the Americas. Based on previous research in Mexico, we expected to find differences between the families related to familiarity with middle-class ways of organizing learning (associated with increased schooling) or familiarity with LOPI. Instead, we found that children in all families were helping at home and that when parents spoke about the goals and values they hoped their children remembered, they consistently spoke about the importance of community, family, and respect in a pattern that is consistent with the ideas of LOPI regardless of increased school experience. We explored the idea of resilient cultural practices in immigrant communities and the development of a repertoire of cultural practices, drawing on multiple traditions in different situations. This contributes to the idea that different cultural forms of organizing teaching and learning need not be mutually exclusive. It also supports the idea that efforts aimed at continuing historical cultural traditions can maintain these cultural practices over generations, even in the case of migration and increased participation in other cultural institutions (like school).

## 1. Learning by Observing and Pitching In to Community Activity

This study examined the interviews of 20 parents of third-grade children in immigrant Latine families in the U.S. The interviews focused on children’s helping at home, the parents’ goals for their children, and the values they hoped to instill in their children. We situated this study within the theoretical framework of Learning by Observing and Pitching In (LOPI), a cultural pattern of organizing children’s lives and learning in many communities of Indigenous heritage of the Americas, where children are incorporated into family and community activities from a young age (Rogoff & Mejía-Arauz, 2022).

LOPI is a way of organizing learning that is consistent with traditions of Indigenous and Indigenous-heritage communities of the Americans, where children are present and included in community activities and are expected to observe and pitch in when ready (Mejía-Arauz et al., 2022; Rogoff & Mejía-Arauz, 2022). This contrasts with how children’s lives have often been organized in middle-class communities where, typically, children contribute in more limited ways and dedicate more time to child-focused activities in preparation for future contributions (Coppens et al., 2016; Coppens & Rogoff, 2022; Morelli et al., 2003). Participation in the community with mutuality and collaboration is key to LOPI (Rogoff & Mejía-Arauz, 2022), and, as such, parents report that it is important to learn and practice the skills necessary to live and work together with considerateness in social “*convivencia*” or togetherness (Aceves-Azuara et al., 2024; Sánchez-Suzuki & Colegrove, 2022).

## 2. Change and Continuity in Cultural Practices

This study also addressed the issue of cultural change and continuity in LOPI practices in immigrant communities. In many previous studies examining practices in LOPI, maternal schooling has often been used as a proxy for experience with LOPI. In these previous studies, families with many years of school experience were often assumed to have less experience with the practice of including children in family and community work. There are compelling reasons for this assumption. One important reason is how school, in and of itself, separates children from families and places them in age-segregated environments. This separation curtails children’s opportunities to work together with multiage community members (Coppens et al., 2016; Rogoff et al., 2003).

Relatedly, decades of research have pointed toward the idea that increased maternal schooling changes the ways in which children learn in the next generation. This change is assumed to be partly based on the idea that with increased schooling, many mothers behave in ways common to school. Studies have found changing patterns of collaboration (Chavajay & Rogoff, 2002; Correa-Chávez, 2016; Mejía-Arauz et al., 2007), attention (López et al., 2012; Silva et al., 2010; Rogoff et al., 1993), and talk (Chavajay & Rogoff, 2002; Correa-Chávez, 2016; Laosa, 1980)—among other changing patterns—with increased maternal schooling among children in the next generation. However, researchers have also argued that schooling is not by itself the “active ingredient” in cultural changes. Scholars have pointed out that there are many changes associated with increased schooling, including smaller family size, increased urban experience, and familiarity with new languages and customs (Rogoff & Angelillo, 2002). Even when changes have been observed in the patterns of the children whose mothers have more experience with school, sometimes the children’s patterns are similar to the patterns found with European American children and sometimes the patterns are different from the patterns of European American children (López-Fraire et al., 2024; Silva & Rogoff, 2021).

## 3. Children’s Helping at Home and Parental Goals

With regard to helping, interviews with parents and children in Mexico have shown that in communities with Indigenous heritage, children tended to collaborate in household work often, stemming from their own initiative (Alcalá et al., 2014; Coppens et al., 2014). However, children from families with extensive schooling (or from non-Indigenous communities in Mexican cities) were minimally involved in household and collaborative work (Coppens et al., 2014; Mejía-Arauz et al., 2015). In interviews, parents in a P’urhépecha (Indigenous Mexican) town explicitly linked children’s helping at home to the goal of teaching children how to work in the community as a way of instilling life skills but also the skills necessary for being a member of a community by working (or co-laboring) together and, thus, learning crucial skills of community and family collaboration (Mejía-Arauz et al., 2015). One of the key goals of P’urhépecha child-rearing is for children to “learn how to work” (Keyser Ohrt, 2015). However, this goal is not the same as developing a strong work ethic by itself; rather, it is a practice of working together with others *and* learning skills that will help them in the future. Both parts were present when P’urhépecha parents talked about children’s work and helping (Mejía-Arauz et al., 2015).

In contrast, in middle-class European American communities, children’s helping is often framed as getting in the way of real work (Coppens & Rogoff, 2022). Among middle-class parents in the United States and Mexico, a common reason given for restricting children’s helping in family work is the need to focus on school as children’s primary form of work (Alcalá et al., 2014; Coppens et al., 2016). This framing resembles the pattern of separating children from productive community activities and teaching children for eventual community participation that is seen in the Assembly Line model of education present in many U.S. schools (Rogoff et al., 2003). In these cases, children’s exclusion from household helping and family work is also tied to parental goals, that of school success with eventual participation in community work years down the line.

However, as previously mentioned, in communities familiar with LOPI, helping at home and community participation are linked, and the cultural importance is apparent and valued just as schooling is valued. In one study, some P’urhépecha parents with extensive school experience indicated some ambivalence regarding the role of schooling in children’s family life. In these interviews, parents reported wanting school and professional success for their children but at the same time lamented the limited time children could devote to family (especially family work) because of school obligations. This awareness led some parents with many years of schooling and professional occupations to make a concerted effort to include family helping practices with their children since these practices could easily be abandoned in busy lives (Mejía-Arauz et al., 2015). Similarly, in the same community, some Indigenous teachers reported making special efforts to incorporate these helping values into the school curriculum in programs focused on Indigenous education (Keyser Ohrt, 2009; Meixi et al., 2022). Thus, although many studies have found differences associated with increased maternal schooling in the children of the next generation, there are also studies where researchers have not found differences related to increased schooling, which is the topic we turn to next.

## 4. Resilient Cultural Practices

People make decisions regarding the practices they engage in for many different reasons; there are no one-size-fits-all models with regard to cultural practice and change (Rogoff, 2003). Sometimes these decisions are deliberately considered, as with the Indigenous heritage teacher’s decisions reported above, and sometimes these decisions may be less deliberate. We refer to resilient cultural practices to describe established and ubiquitous practices that are consistent with LOPI and persist, often despite institutional efforts to discourage them or promote the use of contradicting practices. For example, in a study examining the use of “instructional ribbing” to orient children to the social dimensions of group interactions, European American mothers overwhelmingly disapproved of the practice, whereas Mexican-heritage mothers with assumed LOPI experience (using schooling as a proxy) overwhelmingly approved. However, among Mexican-heritage mothers who had high levels of schooling (and were assumed to have less experience with LOPI), half of the mothers reported approval of the practice and half reported disapproval. Yet, even when some of the Mexican-heritage mothers reported disapproval of this practice, they playfully engaged in it with their children while interacting with the interviewers and their child, indicating the unconscious yet resilient nature of some cultural practices (Silva & Rogoff, 2021).

There have been studies where differences might have been expected between immigrant cultural groups (based on schooling) with regard to helping, but these differences were not found. Ruvalcaba and colleagues (2015) examined a situation where children could request help from an adult who was already busy but present with a child who was building a toy. As expected, there were differences between the children of European American-heritage and those of Mexican heritage, with European American children more commonly interrupting the adult’s activity, and Mexican-heritage children more commonly avoiding interrupting the adult’s activity. However, what was surprising based on a related study on attention using the same data (where schooling differences were found between children of Mexican heritage based on maternal schooling; López et al., 2010) was that there were no differences between the Mexican-heritage children with regard to patiently waiting to ask for help. Both groups of Mexican-heritage children showed *respeto* for the adult, which the authors defined as “a form of caring that can be conveyed through consideration and mutual participation” (Ruvalcaba et al., 2015, p. 188).

In addition to avoiding interruption, the study also found that how the children requested help did not differ between the children of Mexican heritage, but both groups differed from European American children. European American children’s requests for help were more often made verbally, compared to Mexican-heritage children’s requests for help, which were more often nonverbal and unobtrusive. This pattern of results is similar to the pattern found among Indigenous P’urhépecha children in Mexico who, when they had to collaborate on a game but were not allowed to talk, used subtle means of communication that were close to the body and unobtrusive. In the same study, there were also no differences between the groups based on maternal schooling in how they used subtle forms of communication in collaboration (Correa-Chávez & Mejía-Arauz, 2024).

Similarly, when asked to comment on recorded videos of everyday interactions in early childhood classrooms in Texas, Latine parents overwhelmingly reported the importance of children learning to help one another as foundational to being able to live with other community members in respectful *convivencia*, which entails harmonious community participation and interaction (Sánchez-Suzuki & Colegrove, 2022). This sentiment was reported by parents regardless of years of school experience (participants ranged from sixth grade to master’s degrees). The parents framed *convivencia* as an important pedagogical goal in schools where children must learn how to be social beings along with others and practice those skills. Overwhelmingly, the parents in that study were supportive of school efforts to encourage *convivencia* by providing children with the opportunity to help one another in the classroom.

Even when children are discouraged from helping one another in the classroom, many persist in doing so, as this example from a Mapuche School in Chile illustrates:

The teacher points to one of the children who has not finished their exercise and tells them to solve the problem on the whiteboard, but another child responds by saying “…I’ll go, because he hasn’t finished yet…”, but the teacher refuses. Clearly the boy at the whiteboard does not know how to solve the exercise; he is standing in front of the board doing nothing, with the marker in his hand, and [at] the same time looking at his classmates. At that point, several children offer to help, but again the teacher refuses. Nevertheless, another boy goes to help by showing him a notebook with the solved exercise. The teacher reprimands them and tells the entire class they are not to help each other with their work.

(Alarcón et al., 2022, pp. 8–9)

Across many aspects of life, there are indications that these cultural values are resistant in immigrant communities from Indigenous areas of Mexico and Latin America. In discussing issues of identities within Indigenous institutions and spaces, Urrieta (2017) reflected on the importance of community ties (*convivencia* and *communalidad*) in how people with Indigenous histories can recognize one another through communal action rather than racial labels,

Recognition of Indigenous peoples by other Indigenous people, especially of the connected and reciprocal relations of *comunalidad* (Sánchez-López, 2017; Rendón Monzón 2003), or “being a good family and community member”, is one of the most fundamental aspects of Indigenous recognitions.

(Urrieta, 2017, p. 3)

## 5. Building Repertoires of Practice

As communities change, some of these resilient cultural practices may become a part of children and adults’ repertoires of practice. Repertoires of practice are a set of diverse cultural practices that people can draw from in different cultural situations that call on different forms of participation (Gutiérrez & Rogoff, 2003). Children and adults can develop and recognize cultural practices that draw on multiple traditions in different situations. When shown videos, Mexican-heritage 9–11-year-old children in the U.S. could recognize the nonverbal forms of helping that are common in LOPI and discuss how this form of helping was different from helping in school more often than European American 9–11-year-olds (Roberts & Rogoff, 2012). Similarly, elementary school children showed they were aware of the differences in expected behavior tied to cultural expectations. When U.S. Mexican-heritage children (from highly schooled families) were engaged in a school-like task, they were less likely to spontaneously help an adult compared to when they were engaged in work around the house, where they provided help more readily (López-Fraire et al., 2024). And, as previously mentioned, in communities that have extensive experience with school and with LOPI, there is variability in terms of the cultural patterns (Correa-Chávez & Mejía-Arauz, 2024; Ruvalcaba et al., 2015; Silva & Rogoff, 2021), pointing to Urrieta’s (2017) idea on the strength of these communal values and practices and possibly indicating that, in many of these communities, “what is often not ‘seen’ is the *comunalidad* of our everyday life” (p. 3).

## 6. The Current Study

This study expands on previous studies on children’s helping at home within the LOPI paradigm by examining children’s reported helping behaviors within Mexican immigrant communities in the United States and linking them to resilient cultural values that are apparent when caregivers were asked about the goals and aspirations they had for their children. The answers to these questions point to a set of values and practices that are different from what has been found in middle-class Mexican communities in Mexico (Alcalá et al., 2014; Mejía-Arauz et al., 2015). This new pattern adds to our understanding of how LOPI practices persist and adapt across generations and migration. We examined how these families may be building a repertoire of practices where children’s helping at home, the development of skills at work (which includes family and community), and school success are seen as equally important parts of childhood as opposed to competing with one another.

## 7. Methods

### 7.1. Participants

Twenty parents (mostly mothers) of third graders were interviewed as part of a larger study that examined children’s collaborative learning interactions. The age of the children of focus ranged from 8 to 10 years (*M* = 9.2; 10 girls and 10 boys). The mothers’ ages ranged from 26 to 54 years of age (*M* = 37.3), and the fathers’ age ranged from 29 to 47 years of age (*M* = 37.4). All interviewees (except for one) were from Mexican immigrant families: most parents were immigrants themselves (16), and some were children of immigrants (4). Eight mothers had less than 12 years of formal schooling (*M* = 7 years), which we refer to as basic schooling in the Results Section, and 12 mothers had 12 or more years school (*M* = 12.7), referred to as high schooling in the Results Section. One family was from Guatemala and was included because studies have found similar practices related to LOPI. Mothers’ occupations included homemaker, custodian, self-employed cleaning houses, forklift driver, medical assistant, customer service, and college student. Fathers’ occupations included contractor, horse trainer, painter, construction worker, gardener, factory or warehouse work, custodian, dock worker, and electrician. On average, families had 3.3 children in the home, and the child of focus was the oldest child in 6 families, a middle child in 9 families, the youngest child in 4 families, and was an only child in 1 family (primary caregiver was the grandmother). In 7 of the families, parents spoke both English and Spanish; in 13 of families, parents spoke primarily in Spanish. Six of the families mentioned having recent ties to an Indigenous language (e.g., Tarasco/P’urhépecha, Nahuatl, and Q’anjob’al). Parents received a gift card as compensation for their participation.

### 7.2. Measures

All study procedures were approved by the university’s Institutional Review Board. The semistructured interviews focused on family education history, parental goals for their children in the long term, valuable lessons, expectations for helping, and cultural values or traditions that were important to them. The questions were adapted from previous studies examining children’s helping at home within a LOPI paradigm (Alcalá et al., 2014). First, parents were asked demographic questions about family members living at home (age, sex, languages spoken), and then a series of questions focused on views on schooling over generations starting with the parents’ own parents (“What were your parents’ ideas about sending you to school?”) then moving on to their own (“What about you, what do you think about sending kids to school?”; “Do you feel there are activities that help promote your child’s learning in school?”). From there, the interview moved to broader questions about what was important for their children to learn to become the person they would like them to be in the future (“What life lessons or teachings would you want your child to remember you by?”; “In 15 to 20 years what do you imagine your child will be doing in life?”; “What do you think your child is learning now that will help them get to where you want them to be in the future?” “Are there any important cultural traditions or customs that are important to you or your family?”).

Questions then moved on to the child’s everyday activities with a focus on children’s help at home: “Does your child have set activities they do when they get home from school?”, “Does your child have chores or activities they do to help out at home?”, “Are these chores an obligation (something they know they must do) or do they receive an allowance for helping at home?”, “Does your child accompany you or your spouse to work at all?”. Parents were also asked about how they spent the weekends with their child and family. Embedded throughout the conversation, parents also spoke of other important life lessons and cultural values or traditions that were important to them.

### 7.3. Procedure

After their children participated in a larger study examining collaboration, a follow-up semistructured interview was conducted with parents in their home. Interviews were conducted by a bilingual/bicultural research assistant in the parents’ language of choice (English or Spanish), and audio was recorded for analysis. The duration of each semistructured interview ranged from 15 to 30 min.

### 7.4. Data Coding

All audio recordings of the interviews were transcribed by research assistants. Our first step was to count instances of help from the children, and, finding that this was extremely common among all families, we moved to grounded theory content analysis (Sáldaña, 2016) where both authors carefully read each transcript in its original language. We independently coded 25% of the interviews to identify common themes in the interviews. From these, we identified the major themes as children’s helping expectations, views on schooling, and important life lessons. After agreeing on the codes, both authors coded 100% of the data. The themes were coded wherever they occurred in the interview (not just in response to a particular question). We documented the codes on a spreadsheet summarizing the main theme for each of the answers related to the topics (e.g., children’s helping, important life lessons, etc.). Together, we reviewed the codes to identify any discrepancies and identified subthemes that emerged within each of the main themes. Overlap was present 97% of the time, and, in the few instances where there was disagreement, it was due to oversight.

## 8. Results

### 8.1. Result Map

The first step of our analysis involved looking for differences between the families that had 12 or more years of schooling (“high schooling” in quotes below) and those who had fewer years of schooling (“basic schooling” in quotes below). To our initial surprise, we found no differences between families based on family schooling history. Therefore, our analysis focused on finding the patterns that were consistently showing up in how the parents spoke about their children’s helping at home and the future educational and life goals they had for their children within a LOPI framework. We start by presenting the results for helping, highlighting how widespread the practice and expectation of help was in the interviewed families and how it was not seen as inconsistent with school and academic success. Rather, most of the families emphasized the importance of hard work in both endeavors and often linked hard work to issues of care and respect for others in the family and community. We report on these issues and tie them to what we see as resilient practices of valuing community and family.

### 8.2. Helping at Home and Other Activities

#### 8.2.1. High Participation in Household Work

All but one family talked about household work. Out of those, all but one mother reported their child participated in household work. Work included taking care of their own space (e.g., putting toys away, making their bed, cleaning their room), helping with common spaces (e.g., clearing the table, helping with laundry, doing yardwork, taking out the trash), helping family members who needed help (e.g., helping care for sick grandmother) and going to work with parents to help with their jobs (e.g., helping mom at a swap meet stand). When reporting on the household work that the children engage in, one mother reported, “*ellos recogen su ropa y la llevan a la lavandería, recogen juguetes, limpian su cuarto*” (they pick up their clothes, take them to the laundry mat, pick up their toys, clean their room, 40-year-old mother of third-grade girl, basic schooling).

#### 8.2.2. Child Help Is Expected

The majority of mothers (61%) reported that the children did not receive a reward for chores. Furthermore, of those mothers, 64% stressed that helping was expected and a responsibility that their children had. For example,

*Antes de ir a la escuela, tienen que dejar recogido su cuarto y tendida su cama. Fines de semana, me tienen que ayudar a sortear la ropa para ponerla a lavar… pienso que es algo que le ayuda tener una disciplina y que la ve servir para bien de ella porque… yo siempre, y mi papa siempre lo dijo… Que el saber no estorba, ayuda. Entonces el trabajo es bueno, siempre. No le puedo poner un quehacer que yo sé que ella no va a poder hacer.*

Before they go to school, they have to pick up in their room and make their bed. On weekends, they must help me sort the clothes to put it in the wash…I think that it is something that helps her have discipline and is going to help her for the good because…I always, my dad always said…knowing how doesn’t hinder, it helps. So work is good, always. I won’t give her anything that she won’t be able to do (40-year-old mother of third-grade girl, high schooling).

Just like the mother above explained, many mothers stressed the importance of knowing how to do things in the future. For example, “*Es un trabajo que tiene que enseñarse para su futuro*” (it is work that she has to learn for her future, 44-year-old mother of third-grade girl, basic schooling). Another mother emphasized the multiple roles tied to being part of the household and the importance of being an active participant and contributor to what needs to be achieved, even at a young age:

*Sí, es su trabajo. Tienen que ayudar en la casa porque ellas viven allí y les digo que nadie tenemos una sirviente y poco a poco tienen que aprender a limpiar. El día que yo me muera, se van a pudrir en la casa si no hay quien les limpie y tienen que ensañarse desde ahorita.*

Yes, it is their job. They have to help at home because they live there, and I tell them that none of us have a servant and little by little they have to learn to clean. The day that I die, they’re going to rot at home if they don’t have someone to clean for them, and they have to learn now (42-year-old mother of third-grade girl, high schooling).

In an effort to describe the importance of her sons observing their dad’s work, one mother reported, “*en veces se pone (su esposo) a cambiar una llanta y les digo, hijos fíjense”* (at times he [her husband] changes a tire and I tell them, ‘kids, watch, pay attention’; 40-yer-old mother of third-grade boy, basic schooling). Not only did she stress this as important for them helping their father but also for knowing how to perform different kinds of work for their future.

### 8.3. Reports of High Educational Aspirations

All parents (100%) reported high educational aspirations for their children. It often overlapped with the importance of hard work, and some parents stressed education as the most important goal for their children. One mother reported that she wants “*que le echen ganas al estudio. No tengo otra enseñanza, pero esa. El estudio es su futuro*” (that they try hard in school. I do not have another lesson but that one. School is their future; 42-year-old mother of third-grade girl, high schooling). When asked about the importance of the university, another mother reported, “*Sí claro que sí…Creo que es de las otras herencias más grandes que padres le dejan a los hijos… el estudio*” (Yes, of course it is [important]…I think it is one of the other biggest inheritances that parents can leave their children…their studies; mother of third-grade boy, high schooling, age unknown).

When parents were asked about how they imagined their children in 15–20 years, all parents reported that they imagined them either still in a university or having completed their schooling and working in a field that they enjoyed. For example, one mom reported, “*Hay que se reciban, que estudien, que estén graduados, y que estén trabajando en una cosa que les guste*” (that they get a degree, that they go to school, have graduated, and that they are working in something that they like, 40-year-old mother of third-grade boy, basic schooling). Further, a couple of mothers indicated that they understood that interest and success in school cannot be forced. For example, one mother reported, “*Me gustaría que estudiaran. Para mí, pero depende de cada persona o sea no los puedo obligar tampoco si no les gusta, solo yo prefiero que estudien porque la educación para mi es bien importante*” (I would like for them to go to school [college/university]. For me, but it depends on the person because I can’t force them either, but I would prefer for them to go to school because education is very important for me; mother of third-grade girl, basic schooling, age unknown).

### 8.4. Important Lessons in Life

#### 8.4.1. Importance of Working Hard Most Common Response

When parents were asked about lessons that they want their children to remember them by, the most frequently reported response was that they wanted them to work hard in school and in life toward their goals (reported by 80% of parents). For all, part of the idea of hard work overlapped with school as one mother reported, “*Yo quisiera que ellos estudiaran lo mas que se puede, que ellos fueran alguien en la vida*” (I would want for them to continue with school as much as possible, for them to be somebody in life; 40-year-old mother of third-grade girl, basic schooling).

More specifically, among those parents, 44% of them reported that they wanted their child to be someone in life (like the mother above) or to not have to struggle like them (the parents). Another mother explained, “*Mas que nada lo que mas desea uno como padre, que se lleguen a graduar, que lleguen a hacer algo, para cuando estén grandes no estén como uno*” (More than anything, what one desires as a parent is that they graduate, that they are able to do something, that when they are older, there are not like us, mother of third-grade boy, age unknown, basic schooling).

#### 8.4.2. Respeto as a “Way of Being” a Central Theme

Another common response for important lessons was the value of *respeto* as a way of being/way of life, which was reported by 60% of families. One mother reported:

*Yo pienso que …el respeto y el valor de que a veces no es tan importante las cosas. Que más importante, que haiga respeto y amor y eso te lleva a ser, a lo mejor, un buen estudiante y no necesitas tener mucho dinero, pero si tienes respeto por los demás y valoras lo que los demás hacen por ti, agradecimiento, te lleva muchas cosas buenas*

I think that…respect and the value that sometimes (material) things are not that important. The most important thing is that there is respect and love and that will maybe make you a good student and you do not need to have a lot of money, but if you have respect for others and you value what others do for you, gratitude, it can bring many good things (32-year-old mother of third-grade boy, high schooling).

Among other important lessons, *respeto* was also mentioned by the following mother who stressed its centrality, “*que tengan valores y que sepan respetar, que sepan ser honestos y que se ensañen a trabajar, a luchar por sus objetivos, sus metas, lo que ellos quieran*” (that they have values and now how to be respectful, that they know how to be honest, and that they learn how to work, to fight for their objectives, their goals, what they want, 40-year-old mother of third-grade girl, high schooling). When *respeto* was mentioned, links were often made between the interconnected values around being a good person, working hard, honesty and integrity, and respect for others.

#### 8.4.3. Strong Desire to Continue with Cultural and Family Traditions

Another common lesson reported by the parents was their desire to continue with cultural and family traditions (reported by 55% of parents). For example, one mother expressed,

*Pues yo pienso que la enseñanza más importante es el respeto y que ellos aprendan a conocer otras diferentes culturas de mi México. Las culturas y costumbres también que tenemos aparte… porque ahorita están muy pequeños para poder entender… demasiado todo lo que nosotros tenemos de costumbres y tradiciones.*

Well, I think that the most important lessons are respect and for them to learn to recognize other different cultures of my Mexico. The customs and traditions that we have apart from…because they’re currently too young to understand…all that we have in terms of customs and traditions (mother of third-grade boy, age unknown, high schooling).

When speaking about the changes in traditions after migrating to the United States, one parent mentioned that, “*tratamos de enseñar a menos nuestra cultura*” (we try to teach our culture at least) to our children (32-year-old mother of third-grade boy, high schooling), highlighting the difficulty or clash in values often felt by Latine immigrant parents. Parents tended to highlight the extra effort needed to keep certain customs and traditions alive.

Among those traditions, practices and celebrations tied to their faith/religion were common. For example, when explaining an important custom for her, a mother mentioned:

*Otra costumbre que no apunté…que no anotó allí es ir a misa los domingos y es lo que he tratado, que no se pierda porque en parte les ayuda para que se arrimen más a lo espiritual y que no anden desbalagados en cosas de drogas y eso.*

Another custom that I didn’t jot down…that you didn’t make note of is going to mass on Sundays, and I’ve tried to not lose that because, in part, it helps them spiritually and to not get on the wrong track with drugs and that sort of thing (42-year-old mother of third-grade girl, high schooling).

#### 8.4.4. Efforts for *Convivencia*

Another value or lesson commonly reported by parents that they want their children to remember is that of *convivencia* or togetherness/participating in activities with others. Slightly less than half of the parents (45%) expressed the importance of doing things together. When explaining what their children do on weekends, one mother explained,

*Nos vamos al futbol los domingos, juegan. Nomás nos levantamos, nos arreglamos, y nos vamos y pasamos allí la tarde. O nos invitan a comer las parejas de los mismos compañeros del juego, o nos la pasamos tranquilos, y los sábados si nos invitan a un lado, pues vamos todos.*

We go to soccer on Sundays, they play. We just get up, get ready, and we spend the afternoon there, or the other parents will invite us to eat, or just have a relaxing time, and, if on Saturdays, we get invited somewhere, we all go (40-year-old mother of third-grade boy, basic schooling).

The mother emphasized establishing a community with other families in the soccer team and the intentionality in doing things together. The same mother also explained simply spending time together as a family inside the home, “*vemos la televisión juntos entre semana, vemos las novelas en la tarde y me acompaña…y como el fin de semana, los sábados vemos programas (de) imitadores de música de artistas*” (We watch television together during the week, we watch soap operas in the evening and he joins me…and like on weekends, Saturdays we watch programs [about] music star imitators)”

There was also overlap with *convivencia* and participating in religious practices like attending mass together as a family or celebrating religious holidays. When describing the important traditions in her family, one mother mentioned, “*la navidad siempre que nos juntamos y convivimos…pero que uno traiga desde antes, pues es eso que abrazan al niño dios, que se lo pasan, rezan antes de comer*” (We always spend Christmas together and *convivimos*…but what one brings from before is to cradle baby Jesus, pass him around, pray before eating, 40-year-old mother of third-grade girl, high schooling). Similarly, another mother described efforts to *convivir* as family on a typical weekend,

*el sábado… por lo regular despiertan tarde, ven películas, y prácticamente a veces salimos muy poco el sábado que sea a comprar porque su papá trabaja el sábado, pero el domingo prácticamente vamos a misa en la mañana y después de misa los llevamos a desayunar o al parque.*

On Saturday…. they usually wake up late, they watch movies, and we go out very little on Saturday, like to shop because their dad works on Saturday, but on Sunday we usually go to mass in the morning, and after mass, we take them to breakfast or to the park (mother of a third-grade boy, age unknown, basic schooling).

In a follow-up question, the mother stressed that she, her husband, and children go out together on Sundays, highlighting the active effort to *convivir* as a family on the day that everyone is available. This theme of finding time for togetherness was reported in nearly half the families. The percentage of families speaking to each of the reported themes is presented in Table 1.

## 9. Discussion

The qualitative findings in this study point toward the importance of a set of values that emphasize being part of a family, community, and a contributing member of multiple spaces, with children’s work in families being a key activity through which families impart important values and lessons. Rather than pitting school and family work as oppositional, families stressed the importance of hard work in helping in the home, which they tied to children’s overall success and competence in the future. All families stressed high educational aspirations for their children and emphasized the importance of such practices for reaching their educational goals. At the same time, parents specifically stressed the importance of their children working hard to reach their goals (at home and at school) and linked this work to respecting others, continuing with cultural traditions and customs as well as *conviviendo* in family and community spaces.

### Centrality of Helping in Family and Repertoires of Practice

In some studies within the LOPI paradigm, differences have been found between groups from the same community that differ in cultural experience with the institution of school (Chavajay & Rogoff, 2002; Mejía-Arauz et al., 2005; Mejía-Arauz et al., 2007). Initially, based on that previous research and research on children’s helping at home comparing children in middle-class Mexican communities with children in Indigenous heritage communities (Alcalá et al., 2014; Mejía-Arauz et al., 2015), we expected to find differences between the Mexican immigrant families with regard to children’s helping at home and with parents’ discussion of their goals and aspirations for their children. However, in our study, we were surprised by the similar themes reported by the parents regardless of schooling experience. As Ruvalcaba et al. (2015) reported:

To our surprise, the two Mexican backgrounds—those whose families had extensive Western schooling experience and those whose families likely had experience with Indigenous-American practices—did not differ significantly […] We examined the data upside down and backwards to check the surprising lack of differences.

(p. 195)

However, we came to understand that this lack of difference reflected a pattern that was common in studies where children’s participation in family or community was central. Our results were similar to those of Latine parents in the United States (whose schooling ranged from elementary to graduate school), who overwhelmingly agreed on the importance of children developing the skills to achieve harmonious relationships in groups when discussing children’s learning in school (Sánchez-Suzuki & Colegrove, 2022); help and participation in family work at home are important ways in which these skills can develop.

In these immigrant communities, there appears to be what Murray et al. (2017) called a “crucial aspect of everyday life” regarding help and work in the family context. Murray and colleagues called it *cariño* in the Chilean Mapuche community in which they work:

*Cariño* stands for what is considered empathy in different societies, in which feelings and actions related to empathy and empathy-like phenomena are indistinguishable. However, the ubiquity and features of *mutual quotidian care and attentiveness* appeared to be crucial aspects of the everyday lives of these women …

(p. 370, emphasis added)

Whether we call it *cariño, comunalidad, convivencia*, or *respeto*, it appears there is an everyday core value that resists change, or, if it is not resistance as such, it allows for multiple frames of reference and interaction to develop as an expansion of people’s repertoires of practice.

The fact that parents can speak to these values so clearly in this study and others (Aceves-Azuara et al., 2024; Mejía-Arauz et al., 2015; Sánchez-Suzuki & Colegrove, 2022) indicates the importance of these values. This centrality might make it easier for children (and families) to develop skills of biculturalism and the understanding that they might act one way in school and another way at home. This conjecture aligns with the findings of López-Fraire et al. (2024), who found Mexican-heritage children from families with extensive schooling were more likely to jump in and help at home (like Mexican-heritage children from families with less schooling) but were less likely to jump in and help in a school-like situation. This also aligns with the finding that U.S. Mexican-heritage children were more likely to identify nonverbal requests for help (compared to European American children), but there was no easy way to talk about this complex set of helping behaviors for the children (Roberts & Rogoff, 2012). In contrast, adults in Mexico were able to elaborate on this idea by using the Spanish term *acomedido*, which loosely translates as being attentive and helping with initiative (López et al., 2012, 2015).

This set of findings, along with previous research, might also suggest that it may be beneficial for parents and children to explicitly identify these helping behaviors as an important cultural value if the goal is to continue participating and building on it. Future research can perhaps help build on the ideas present in this form of helping to encourage children from Indigenous-heritage families of the Americas to continue to use and value these cultural learning practices. The prevalence of family helping practices also demonstrates the extent of agency and initiative often granted to children at home, even as children of color are often denied the opportunity to demonstrate that agency in the school context (Adair & Sánchez-Suzuki Colegrove, 2021). However, as we work to transform schools to grant children more agency in their school life, the helping practices mentioned here may be key for children who come from families that are familiar with LOPI practices.

Further building on the idea of repertoires of practice, this study also contributes to the idea that different cultural forms and values in organizing teaching and learning need not be mutually exclusive. In this study, the parents from families that were more familiar with LOPI expressed both a desire for children to succeed at school *and* encouraged children to contribute to family work at home. Children’s help and initiative in helping are highly valued in communities with Indigenous history and are skills that can be built upon across many contexts: home, community, and school, in a way that fosters agentic school success (even if this is not currently the case in many school contexts; Adair et al., 2018; Adair & Sánchez-Suzuki Colegrove, 2021).

## 10. Limitations and Conclusions

Although this study’s findings fit within a pattern of findings that highlight the centrality of children’s help as a way to emphasize the importance of harmonious participation in community, there are limitations we can address. The findings in this study rely on parental reports of children’s activities. We did not observe children’s participation directly, and thus it is possible that parents’ own feelings of what is important may have influenced their reporting of their child’s helping behavior at home. However, even if this were the case, we think the results are interesting in that the parental reports coincide with other studies’ findings in immigrant communities (López-Fraire et al., 2024; Ruvalcaba et al., 2015; Sánchez-Suzuki & Colegrove, 2022). Yet, the findings contrast with parental reports from middle-class families in large cosmopolitan Mexican cities when parents were asked similar questions about children’s helping at home (Alcalá et al., 2014; Mejía-Arauz et al., 2015). There is scholarship suggesting that immigrant communities conserve traditions that may be changed in the sending countries (Rogoff, 2003). Our own findings regarding the centrality of religious traditions in these families seem to support this suggestion that families are maintaining traditions from their sending countries. Whether this is the case regarding the cultural values around children’s helping at home is a question that requires more study. For example, it is possible that the centrality of community and helping may be especially highlighted in immigrant communities precisely because they are physically separated from previous community help networks by migration.

Another limitation of our study is the small sample size. However, helping behaviors in Latine communities have been found in many studies (Dorner et al., 2007; Faulstich Orellana et al., 2003) including large-scale ones (Fuligni, 2001). In both large-scale and small-scale studies, Mexican-heritage adolescents who engage in helping behavior at home also perform better at school (Dorner et al., 2007; Fuligni, 2001). However, none of these studies (including ours) were specifically designed to examine the question of *why* family helping seems to be beneficial to children and adolescents in Mexican immigrant families. Nonetheless, working within the tradition of LOPI and incorporating the work of scholars working with communities of Indigenous heritage of the Americas, we believe our results suggest that harmonious relationships and care are key aspects of this helping behavior and may thus be important reasons we see this practice continue across generations in immigrant families.

The results from this study help bolster the argument above in that most of the parents themselves independently linked children’s helping at home with school success. These findings highlight a strength that is present in many families with Indigenous histories in the Americas. Attending to these strengths and incorporating them in child-serving contexts (schools, afterschool clubs, museums, etc.) can help encourage fuller participation from parents, as the robustness of the interviews showed the interconnected vision of children’s helping and children’s school success in these parents’ eyes. Thus, by attending to these key cultural values, we can help create more welcoming spaces where children’s agency and children’s helping are recognized, valued, and built upon.

## Figures and Tables

**Table 1:** Summary of main themes and subthemes identified. Subthemes ordered hierarchically.

Theme			Percentage of Families	
Helping At Home and Other Activities				
	Child Participation in Household Work			95%
		No reward for helping at home		61%
			Child help was expected	64%
Reports of High Educational Aspirations				100%
Important Lessons in Life				
	Importance of Working Hard in General			80%
		Some overlap between work/helping family and school		100%
		Wanted children to “be someone in life”		44%
	Respeto as a Way of Being			60%
	Desire to Continue Cultural and Family Traditions			55%
	Efforts for Convivencia			45%

Note: Major themes are underlined, and different levels of sub-themes are indented.

## Data Availability

The data and the materials necessary to reproduce the analyses and to attempt to replicate the findings are not publicly accessible due to ethical considerations and to protect the privacy, confidentiality, and anonymity of the vulnerable populations involved in this study.
